# Cytomegalovirus-Specific T Cells Restricted by HLA-Cw*0702 Increase Markedly with Age and Dominate the CD8^+^ T-Cell Repertoire in Older People

**DOI:** 10.3389/fimmu.2017.01776

**Published:** 2017-12-11

**Authors:** Louise Hosie, Annette Pachnio, Jianmin Zuo, Hayden Pearce, Stanley Riddell, Paul Moss

**Affiliations:** ^1^College of Medical and Dental Sciences, Institute of Immunology and Immunotherapy, Birmingham Health Partners, University of Birmingham, Birmingham, United Kingdom; ^2^Fred Hutchinson Cancer Research Center, Seattle, WA, United States

**Keywords:** cytomegalovirus-specific CD8 T cells, memory inflation, HLA-Cw*0702, aging, immediate-early antigen

## Abstract

Cytomegalovirus (CMV) infection elicits a strong T-cell immune response, which increases further during aging in a process termed “memory inflation.” CMV downregulates the expression of HLA-A and HLA-B on the surface of infected cells to limit presentation of viral peptides to T-cells although HLA-C is relatively spared as it also engages with inhibitory killer immunoglobulin receptor receptors and therefore reduces lysis by natural killer cells. We investigated the magnitude and functional properties of CMV-specific CD8^+^ T-cells specific for 10 peptides restricted by HLA-C in a cohort of 53 donors between the age of 23 and 91 years. This was achieved *via* peptide stimulation of PBMCs followed by multicolor flow cytometry. Three peptides, derived from proteins generated in the immediate-early period of viral replication and restricted by HLA-Cw*0702, elicited strong immune responses, which increased substantially with age such that the average aggregate response represented 37% of the CD8^+^ T-cell pool within donors above 70 years of age. Remarkably, a single response represented 70% of the total CD8^+^ T-cell pool within a 91-year-old donor. HLA-Cw*0702-restricted CD8^+^ T-cell responses were immunodominant over HLA-A and HLA-B-restricted CMV-specific responses and did not show features of exhaustion such as PD-1 or CD39 expression. Indeed, such CTL exhibit a polyfunctional cytokine profile with co-expression of IFN-γ and TNF-α and a strong cytotoxic phenotype with intracellular expression of perforin and granzymeB. Functionally, HLA-Cw*0702-restricted CTL show exceptionally high avidity for cognate peptide-HLA and demonstrate very early and efficient recognition of virally infected cells. These observations indicate that CD8^+^ T-cells restricted by HLA-C play an important role in the control of persistent CMV infection and could represent a novel opportunity for CD8^+^ T-cell therapy of viral infection within immunosuppressed patients. In addition, the findings provide further evidence for the importance of HLA-C-restricted T-cells in the control of chronic viral infection.

## Introduction

Human cytomegalovirus (CMV) is a β-herpesvirus with a seroprevalence of 60–90% depending on geographical location. Although primary infection is typically asymptomatic, the virus establishes a state of persistent infection and CMV remains a major cause of morbidity and mortality in immunocompromised individuals such as the fetus or transplant recipients (1, 2). CMV undergoes periodic episodes of reactivation and CD8^+^ T-cells play a critical role in controlling viral reactivation (3–7). Indeed, CMV infection triggers one of the largest virus-specific cellular immune responses yet determined (8), and an unusual feature is that the magnitude of this response increases further with age in a process termed “memory inflation” (9–14).

HLA-C is a HLA class I haplotype that is expressed at a lower level at the cell surface compared with HLA-A and HLA-B (15). HLA-C can present peptides to virus-specific T-cells but much less is known about CD8^+^ T-cell recognition of peptides restricted by HLA-C (15, 16). HLA-C surface expression is controlled by both transcriptional and posttranslational mechanisms (17) and is also negatively regulated by miRNA-mediated expression (18, 19). Importantly, HLA-C is the major ligand for inhibitory killer immunoglobulin receptors on natural killer cells (NK cells) and as such exhibits a dual role between NK and T-cell recognition (15, 18). This observation is likely to explain why virally encoded immune evasion proteins which downregulate the expression of most HLA class I proteins have evolved to allow relative retention of HLA-C at the cell surface to prevent NK recognition of the infected cell. The HIV-encoded nef protein exhibits this selective pattern of HLA downregulation, and it is noteworthy that polymorphism within the HLA-C locus and the presence of HLA-C-restricted CD8^+^ T-cells have are with reduced HIV viral load and slower disease progression (19–22). HLA-C-restricted T-cells are also important in hepatitis B infection where HLA-Cw0801-restricted recognition of Env171–180 is associated with viral clearance (23).

Almost all studies of CMV-specific CD8^+^ T-cell immune responses have focused on peptides restricted by HLA-A or HLA-B (14, 24, 25). However, CMV also encodes a range of proteins and RNAs that downregulate the expression of HLA class I molecules on infected cells (26–29) although the expression of HLA-C is relatively less suppressed (29–32). At the current time, relatively little is known regarding the magnitude or function of HLA-C-restricted CMV-specific T-cells (11), and these have not been studied in older people.

We investigated the magnitude and phenotype of CMV-specific CD8^+^ T-cells restricted by HLA-C and investigated how these features change during healthy human aging. We show that HLA-Cw*0702-restricted CD8^+^ T-cells specific for three peptides derived from the immediate-early (IE) period of viral replication expand dramatically with age. These can represent up to 70% of the CD8^+^ T-cell repertoire in people over the age of 70 years. These CD8^+^ T-cells exhibit a polyfunctional IFN-γ^+^TNF-α^+^ profile and a strong cytotoxic capacity that increases with age. This information provides new insights into the mechanism of immune control of CMV infection and may also represent novel opportunities for viral-specific immunotherapy.

## Materials and Methods

### Ethics Statement

The recruitment of healthy participants was approved by the Solihull ethics committee, study reference; 14/WM/1254. All participants were adults aged over 18 years, and written and informed consent was taken before participation in accordance with the Declaration of Helsinki. This donor cohort included samples from healthy laboratory personnel, healthy donors from the NHS blood transfusion service, and healthy older adults recruited from the “Birmingham 1000 Elders cohort.”

### CMV Epitopes Studied

Novel HLA-C CMV peptide sequences were kindly provided by Stanley Riddell^2^ or obtained from the literature and described in Table 1. All peptides were purchased from “Peptide2.0” synthesized with crude purity (20–80%), resuspended in 100% dimethyl sulfoxide and stored at −20°C. Novel HLA-C peptides were identified after coculture of RV798 strain infected fibroblasts with PBMCs from seropositive donors (33). The protein antigens to which expanded CD8^+^ T-cells were specific for were identified after coculture with RV798 strains deleted for single CMV genes and loss of IFN-γ production (data not shown). The RV798 strain of HCMV is based on the AD169 strain backbone but carries a deletion of the US2-11 immune evasion proteins (28). The peptide specificity of the CD8^+^ T-cell clones was determined after coculture with COS7 cells transfected with overlapping peptide fragments from the determined cognate CMV protein (Riddell, unpublished data). The HLA restriction of the peptides was identified as either HLA-Cw*0702- or HLA-Cw*1601-restricted using the SYTHPETHI database. This was subsequently confirmed by IFN-γ ELISA (Thermo Scientific) after incubation with EBV-transformed LCL lines expressing a single HLA allele from the donor (HLA-Cw*0702 UL28-derived FRC HLA-restriction data provided in Figure S1 in Supplementary Material).

**Table 1:** List of cytomegalovirus (CMV) epitopes restricted by HLA-C.

CMV protein	Amino acid location	Peptide sequence	HLA-C restriction	Kinetics of expression	Reference
pp65	7–15	RCPEMISVL	Cw1	L	(2)
pp65	341–350	QYDPVAALF	Cw4	L	(2)
Immediate-early-1 (IE-1)	88–96	QIKVRVDMV	Cw6	IE	(2)
pp65	198–206	VVCAHELVC	Cw8	L	(2)
IE-1	305–313	LSEFCRVL	Cw0702	IE	(34)
IE-1	309–317	CRVLCCYVL	Cw0702	IE	(11, 30)
UL28	327–335	FRCPRRFCF	Cw0702	IE	^a^
UL33	120–128	SYRSTYMIL	Cw0702	L	^a^
pp65	294–302	VAFTSHEHF	Cw12	L	(2)
UL24	120–134	YLCCQTRLAFVGRFV	Cw1601	L	^a^

*The CMV protein from which the epitope is derived, the amino acid location of the peptide epitope and the HLA-restriction allele are shown. In addition, the kinetics of expression of the protein during viral replication is indicated as “immediate early” (IE) or “late” (L). The peptide name is denoted by the first three amino acids of the sequence*.

^a^Identified by Riddell et al., Fred Hutchinson Cancer Research Centre.^2^

### Study Subjects

33 Young and 20 older healthy CMV-seropositive donors were included in this study, defined as <70 or >70 years, respectively. Peripheral blood mononuclear cells were isolated from whole blood by density gradient centrifugation (Lymphoprep: Nycomed). DNA for MHC class I typing was isolated from the isolated PBMCs using the “DNeasy Blood and Tissue Kit” from Qiagen according to the manufacturer’s guide. CMV-seropositive donors were HLA typed for MHC class I by PCR-based methods as previously published (35).

### Intracellular Cytokine Secretion Assay and Flow Cytometry Analysis

CD8^+^ T-cells activated by CMV peptides were assessed by measuring the production of IFN-γ, IL-2 and TNF-α *via* flow cytometry after PBMC stimulation. PBMCs from seropositive donors were peptide-stimulated with 1 µg/mL peptide (final concentration) and 1 µg/mL brefeldin A (final concentration) for 6 h. Control PBMCs were stimulated with 10 µg/mL (final concentration) “Staphylococcus enterotoxin B” or remained unstimulated. After incubation, PBMCs were stained with LIVE/DEAD Fixable Dead Cell Stain-APC (Invitrogen), CD3-pacific blue (eBioscience) and CD8-PerCP-Cy5.5 (eBioscience). Cells were fixed in 4% paraformaldehyde followed by permeabilization with 0.5% saponin for 5 min at room temperature (RT) before the addition of IFN-γ-FITC (BioLegend), IL-2-PE (BioLegend) and TNF-α-Pe-Cy7 (eBioscience) for 30 min at RT in the dark. Cells were analyzed on the LSR II (BD Biosciences) and data processed on “Kaluza 1.3” software (Beckman Coulter). The cytokine polyfunctionality of CMV epitope-specific CD8^+^ T-cells was determined using Boolean gating within Kaluza software and analysis using the “Funky Cells” software (36). An average of between 200,000 and 300,000 live lymphocytes events were recorded per sample. The percentage of activated, and therefore specific, cytokine producing CD8^+^ T-cells was calculated as a proportion of the total CD8^+^ T-cell population. In all cases, the background negative control cytokine production was subtracted from the peptide-stimulated tubes.

### CMV-Specific CD8^+^ T-Cell Cloning

Cytomegalovirus peptide-specific IFN-γ producing CD8^+^ T-cell clones were isolated from PBMCs of seropositive donors *via* a limiting dilution assay. PBMCs were peptide-stimulated for 3 h, and peptide-specific CD8^+^ T-cells isolated using the “IFN-γ secretion detection” kit (Miltenyi Biotech) according to the manufacturer’s instructions. Specific CD8^+^ T-cells were plated onto v-bottom 96 well plates in cloning media [RPMI supplemented with 10% fetal calf serum (FCS), 1% human serum (HuS), and 1% penicillin/streptomycin (P/S)] at 0.3 T-cells per well. These were cocultured with γ-irradiated PBMCs and HLA-matched and peptide-loaded γ-irradiated LCLs and left at 37°C for 14 days at 37°C. At day 3, these were supplemented with 60% supernatant from the MLA-144 IL-2-secreting T-cell line (37) and 100 U/mL IL-2. Expanded wells were tested for peptide-specificity by IFN-γ ELISA (Thermo Scientific) according to the manufacturer’s instructions after O/N coculture with peptide-loaded HLA-matched LCLs. Epitope-specific CD8^+^ T-cell clones were expanded *in vitro* in T-cell media (RPMI, 30% MLA, 10% FCS, 1% HuS, and 1% P/S) and restimulated with peptide-pulsed γ-irradiated LCLs and PBMCs every 21 days.

The avidity of CD8^+^ T-cell clones was determined by overnight coculture of 1,000 CD8^+^ T-cells and 1 × 10^4^ LCLs per well on a v-bottom 96 well plate with a gradient peptide concentration in RPMI (supplemented with 10% FCS and 1% P/S) ranging from 50 µM (10^−5^) to 50 pM (10^−11^). Positive recognition of the peptide gradient was determined by IFN-γ ELISA (Thermo Scientific) according to the manufacturer’s instructions.

The avidity of HLA-Cw*0702-restricted CD8^+^ T-cells within donor PBMC was determined as previously described (38). Briefly, 50,000 PBMCs were incubated for 16 h with a peptide gradient ranging from 50 µM (10^−5^) to 50 pM (10^−11^), and peptide-specificity was determined by intracellular cytokine staining for TNF-α. The EC_50_ of the HLA-Cw*0702-restricted PBMC and CD8^+^ T-cell clones was determined in GraphPad Prism 6 by applying a sigmoidal dose response variable slope.

### CD8^+^ T-Cell Recognition of CMV-Infected Fibroblasts

MRC5 (HLA-Cw*0702^+^) were purchased from ATCC and maintained in DMEM media supplemented with 10% FCS, 1% P/S, and 1% glutamine (Q). 1 × 10^4^ fibroblasts/well were then infected with the *AD169* CMV strain at an MOI of 5 and left for 6–72 h at 37°C. At the relevant time point, 10,000 epitope-specific CD8^+^ T-cell clones were added per well, and CD8^+^ T-cell recognition of peptide measured by IFN-γ ELISA after 16 h.

### Statistics

All statistics were computed in GraphPad Prism 6 using non-parametric tests. Specific tests are indicated in figure legends. Statistical significance was defined as * as *p*-value of <0.05, **<0.01, ***<0.001.

## Results

### CMV Peptides Synthesized in the IE Period of Viral Replication and Restricted by HLA-Cw*0702 Stimulate Strong CD8^+^ T-Cell Immune Responses Which Increase Markedly with Age

To determine the magnitude of the CMV-specific HLA-C-restricted CD8^+^ T-cell response, we initially studied 10 peptides restricted through different HLA-C alleles (Table 1). To the best of our knowledge, these epitopes represent all currently defined CMV HLA-C-restricted epitopes. Four peptides were derived from the immediate-early-1 (IE-1) protein and one from the UL28 protein, both of which are synthesized in the IE period of viral replication. By contrast, six epitopes were derived from pp65, UL33, or UL24, which are generated in the “late” stage of viral replication (Table 1).

**Figure 1:**
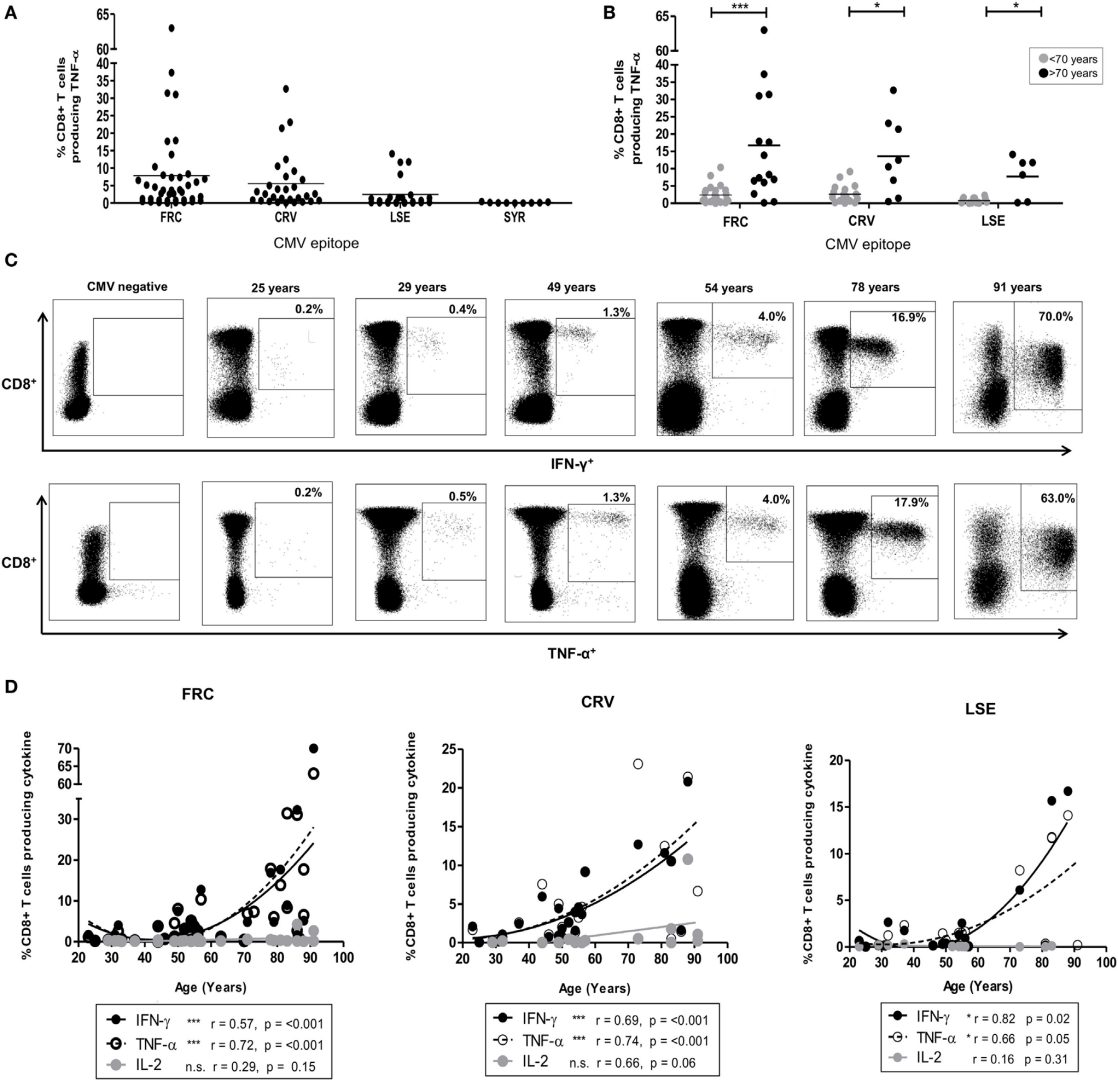
CD8^+^ T-cell response after stimulation by cytomegalovirus (CMV) peptides restricted by HLA-Cw*0702. **(A)** The magnitude of CD8^+^ T-cell TNF-α production in response to peptide stimulation in HLA-Cw*0702^+^ CMV-seropositive donors. Peptide-specific CD8^+^ T-cells were identified after peptide stimulation and intracellular cytokine staining. Each symbol represents the percentage of an individual donor’s total CD8^+^ T-cell pool producing TNF-α in response to the peptide. The peptide name and temporal pattern of expression during the viral lifecycle are shown on the *x*-axis. Horizontal lines represent the mean. **(B)** The magnitude of CD8^+^ T-cell TNF-α production in response to peptide stimulation in HLA-Cw*0702^+^ CMV-seropositive donors aged <70 or >70 years of age. Peptide-specific CD8^+^ T-cells were identified as in panel **(A)**. Each symbol represents the percentage of an individual donor’s total CD8^+^ T-cell pool producing TNF-α in response to the peptide. Gray circles represent donors <70 years of age, and black circles represent donors >70 years of age. Horizontal lines represent the mean. Statistical significance was determined by Mann–Whitney within GraphPad Prism 6 (*p < 0.05 and ***p < 0.001). **(C)** Representative plots of CD8^+^ T-cell cytokine production in six donors following stimulation with FRC peptide. FRC-specific CD8^+^ T-cells were identified as in panel **(A)** to assess IFN-γ (top row) and TNF-α (bottom row) production. CD8 expression is shown on the *y*-axis and cytokine expression on the *x*-axis. The age of the donor is provided above the representative plots. The gating strategy is provided in Figure S2 in Supplementary Material. **(D)** Magnitude of cytokine production following stimulation with peptides FRC, CRV, and LSE shown in donors according to age. Peptide-specific CD8^+^ T-cells were identified as in panel **(A)**. The age of the donors in years is shown on the *x*-axis, and the percentage of the total CD8^+^ T-cell cytokine response is provided on the *y*-axis. Significance was calculated by Spearman’s rank correlation.

A panel of 53 healthy CMV-seropositive donors aged 23–91 years was screened for peptide responses using intracellular cytokine staining (Table 1 and Figure 1A; Figures S3 and S4 in Supplementary Material). The number of IFN-γ, TNF-α, or IL-2-positive CD8^+^ T-cells was then expressed as a percentage of the total CD8^+^ T-cell pool (Figure 1A, gating strategy Figure S2 in Supplementary Material). To examine if the magnitude of immune response was influenced by donor age, we then compared responses within donors aged below or above the age of 70 years. Donors were screened for peptide responses if they expressed the relevant HLA allele of the peptide in question.

CD8^+^ T-cell responses to peptides restricted by HLA alleles *HLA-Cw1/4/6/8/12/16* ranged between 0.02 and 14.2% (Figure S4 in Supplementary Material). A single large response was detected in a donor of 86 years toward the HLA-Cw*1601-restricted YLC peptide (Figure S4 in Supplementary Material). By contrast, striking TNF-α and IFN-γ CD8^+^ T-cell responses were observed to three peptides restricted by HLA-Cw*0702 (FRC, CRV, and LSE, Table 1), and these became the focus of further studies in this report (Figure 1A).

FRC-specific responses were found in 74% (32/43) of HLA-Cw*0702^+^ donors (Figure 1A), and the TNF-α response increased from an average of 2.4% of the total CD8^+^ T-cell pool in donors <70 years to an average of 16.7% in donors above this age (Figure 1B). The IFN-γ production increased similarly from 2.5 to 14.9% while IL-2 responses were low and increased slightly from 0.2 to 0.9%, respectively (Figure S3 in Supplementary Material). Remarkably, the IFN-γ and TNF-α responses against this peptide comprised 63 and 70% of the peripheral CD8^+^ T-cell repertoire respectively in the eldest donor aged 91 years (Figure 1C).

Peptides CRV (39) and LSE (34) are derived from the IE-1 protein and also elicited strong CD8^+^ T-cell immunity which increased in relation to the age of the donors (Figures 1A,B). Specifically, CRV- and LSE-specific responses ranged from 0.01 to 32.7% and from 0.01 to 16.7% of the total CD8^+^ T-cell response and were detected within 80 and 76% of individuals (20/25 and 16/21), respectively (Figure 1A). The average TNF-α response to CRV increased from 2.7% in donors <70 years to 13.6% of the CD8^+^ T-cell repertoire in those aged >70 years (Figure 1B). Corresponding average TNF-α values for LSE-specific responses increased from 0.8 to 7.7% between these two age groups (Figure 1B). As with FRC-specific responses, the IFN-γ response to peptide stimulation also increased with age while IL-2 responses remained comparatively low (Figure S3 in Supplementary Material). Interestingly, CD8^+^ T-cell responses against the SYR peptide derived from the UL33 protein, expressed at a late stage in viral replication (Figure 1A), were relatively weak. TNF-α responses to this HLA-Cw*0702-restricted peptide ranged from 0.05 to 1% of the total CD8^+^ T-cell pool (Figure 1A: IFN-γ and Il-2 responses in Figure S3 in Supplementary Material).

A cross-sectional profile of CD8^+^ T-cell responses against the FRC, CRV, and LSE peptides in relation to age revealed a strong “inflationary” profile (Figures 1C,D) with the most dramatic responses seen in donors aged over 70 years (Figure 1D). As such, this work shows that strong and “inflationary” CD8^+^ T-cell immune responses are observed against three peptides synthesized in the IE period of viral replication and presented through HLA-Cw*0702. Therefore, both the temporal generation of the epitope and allelic restriction are important determinants of the immunodominance of the CMV-specific immune response restricted by HLA-C.

### The Aggregate Response to Immunodominant CMV Peptides Restricted by HLA-Cw*0702 Can Represent over 70% of the CD8^+^ T-Cell Repertoire in Older People

**Figure 2:**
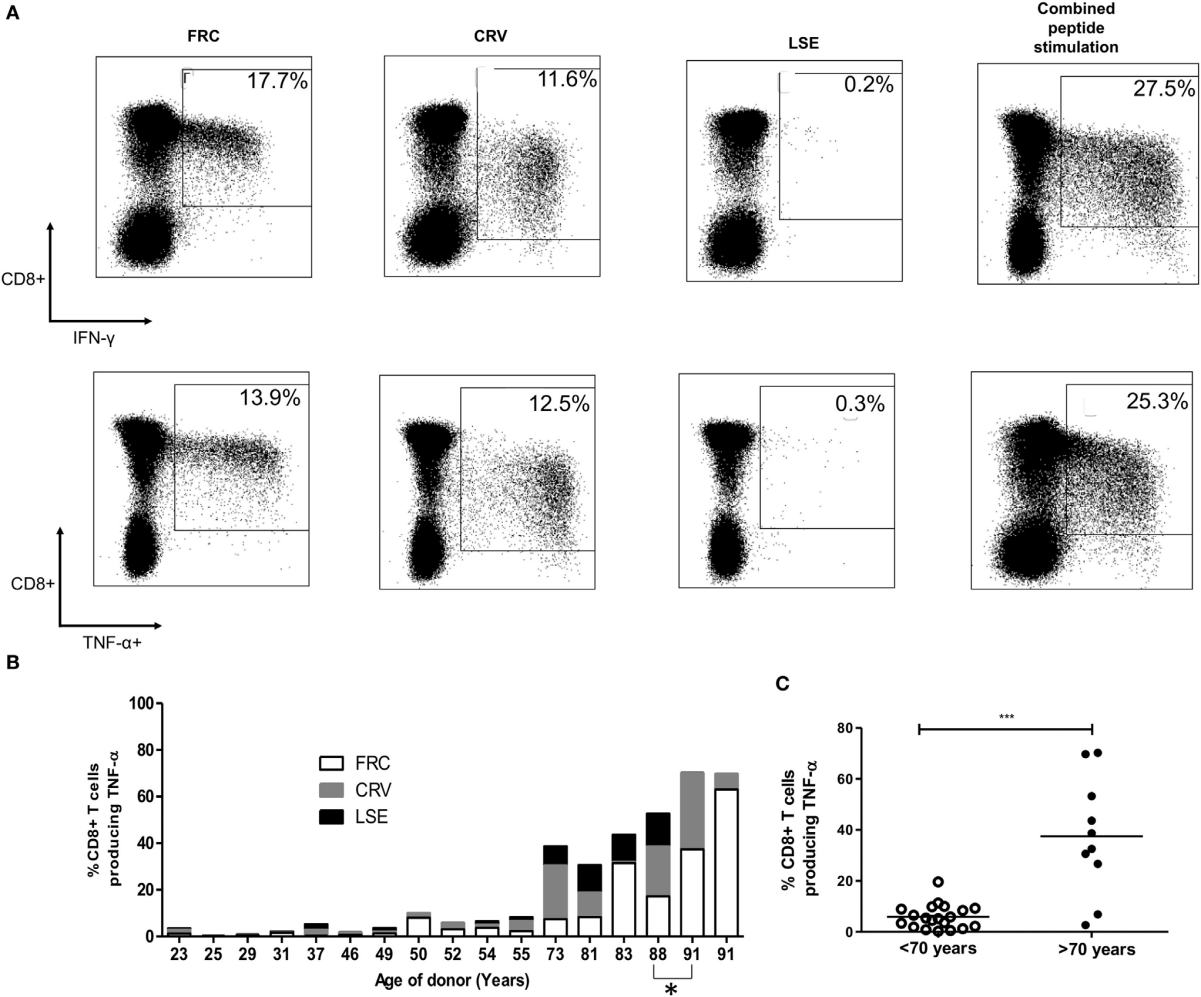
CD8^+^ T-cell responses against cytomegalovirus (CMV) peptides restricted by HLA-Cw*0702 are not cross-reactive, and their aggregate response represents a large percentage of the total CD8^+^ T-cell repertoire within older donors. **(A)** CMV-specific T-cell responses to different peptides do not show evidence of cross reactivity. PBMC from a CMV-seropositive donor aged 81 years was stimulated with either FRC (left), CRV (middle), LSE peptide (right), or all three peptides (far right) before intracellular cytokine staining for IFN-γ (top row) and TNF-α production (bottom row). **(B)** Distribution of individual peptide-specific CD8^+^ T-cell responses producing TNF-α within 16 donors. The TNF-α response to each peptide stimulation is shown as a percentage of the total CD8^+^ T-cell repertoire within individual donors (*y*-axis). The age of the donor in years is shown on the *x*-axis. One donor is included at subsequent time points (88 and 91 years) indicated by *. **(C)** Total magnitude of CMV-specific CD8^+^ T-cell response restricted by HLA-Cw*0702 within individual donors. Each circle represents the aggregate of the FRC, CRV, and LSE-specific CD8^+^ T-cell responses within individual donors as identified in panel **(A)**. White circles represent donors <70 years of age, and black circles represent donors >70 years of age. Lines represent mean, and significant difference was determined using Mann–Whitney tests in GraphPad Prism 6 (***p < 0.001).

To determine the total proportion of the CD8^+^ T-cell repertoire that was represented by HLA-Cw*0702-restricted responses, we combined individual donor peptide responses to determine the total aggregate response against the three epitopes (Figure 2B). The combined TNF-α CD8^+^ T-cell response ranged from 0.31% of the CD8^+^ T-cell repertoire in a 25-year-old donor to 70.3% in the oldest donor of 91 years (Figures 2B,C). IFN-γ responses were comparable whereas IL-2 responses ranged from 0.13 to 11.1% (data not shown). Responses against all three peptides were maintained in most donors, although FRC-specific CD8^+^ T-cells became the most dominant population within the two oldest donors aged 88 and 91 years (Figure 2B).

Importantly, simultaneous stimulation of PBMC from three donors aged 32–81 years with all three HLA-Cw*0702-restricted peptides in combination detected a similar response to the sum of the three individual peptide stimulations, indicating that these responses are not cross-reactive (representative donors shown Figure 2A). This feature was further confirmed by the failure of peptide-specific clones to respond to stimulation with the alternate epitopes (data not shown).

### Inflation of HLA-Cw*0702-Restricted CD8^+^ T-Cell Responses with Age Is Apparent in Prospective Studies within Individual Donors

**Figure 3:**
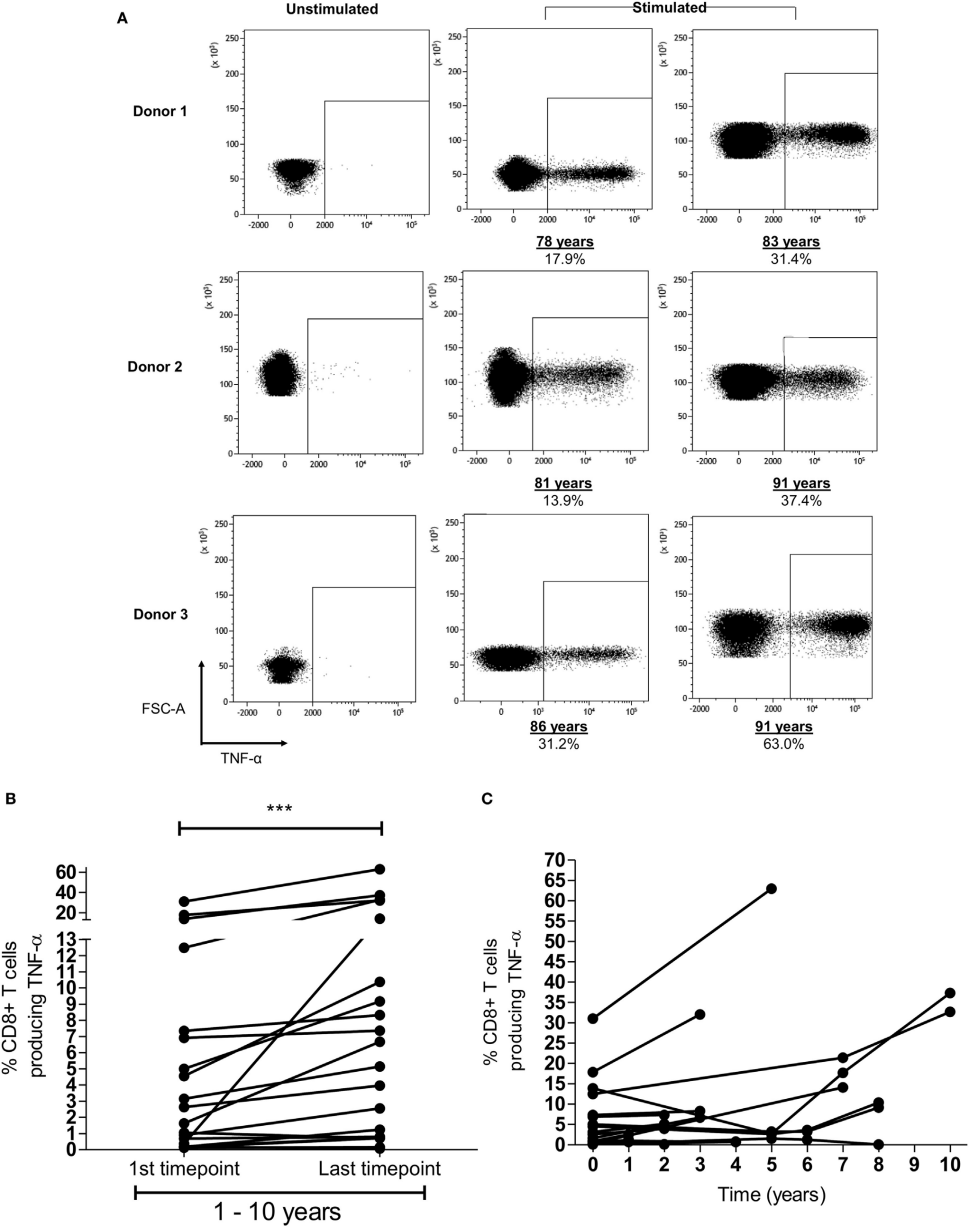
Memory inflation of HLA-Cw*0702-restricted T-cell responses is seen within individual donors. **(A)** Example of memory inflation within donors. FRC-specific CD8^+^ T-cell responses were followed within nine donors using blood samples taken between 1 and 10 years apart. This figure shows the TNF-α response in three donors at the initial and final time points. Percentages represent the TNF-α response to peptide as a proportion of the total CD8^+^ T-cell repertoire. The same number of lymphocytes is demonstrated in the two representative plots at each time point per donor. [Additional time points are provided in panel **(C)**.] **(B)** Summary of longitudinal analyses of peptide-specific CD8^+^ T-cell responses. The initial and last time points (after 1–10 years) for each HLA-Cw*0702-restricted CD8^+^ T-cell response are shown for all nine donors. Statistical significance was determined by a paired Wilcoxon test. **(C)** Representation of all prospective peptide-specific T-cell responses at individual time points within each donor.

In addition to the finding of memory inflation of HLA-Cw*0702-restricted responses in the cross-sectional analysis of the donor cohort, we were also interested to see if CD8^+^ T-cell responses were found to increase in prospective studies within donors (representative FRC-specific CD8^+^ T-cell TNF-α responses provided in Figure 3A). Blood samples were available from nine HLA-Cw*0702-positive donors at time points between 1 and 10 years apart. A significant increase in the CD8^+^ T-cell pool response to the three HLA-Cw*0702-restricted peptides was observed during follow up (Figure 3B). For two donors where four time points were available, fluctuations were observed but the percentage of HLA-Cw*0702-restricted CD8^+^ T-cells were significantly increased over an 8- or 10-year period compared with the initial measurement (Figures 3B,C). The most dramatic increases were observed in donors aged >70 years where, for example, an FRC-specific response comprising 31% of the CD8^+^ T-cell pool in an 86-year-old donor expanded to 63% over a period of 5 years (Figures 3A,C; Figure S5 in Supplementary Material).

### HLA-Cw*0702-Restricted CD8^+^ T-Cells Demonstrate a Cytotoxic Phenotype Which Increases Further during Aging without Evidence of Exhaustion

**Figure 4:**
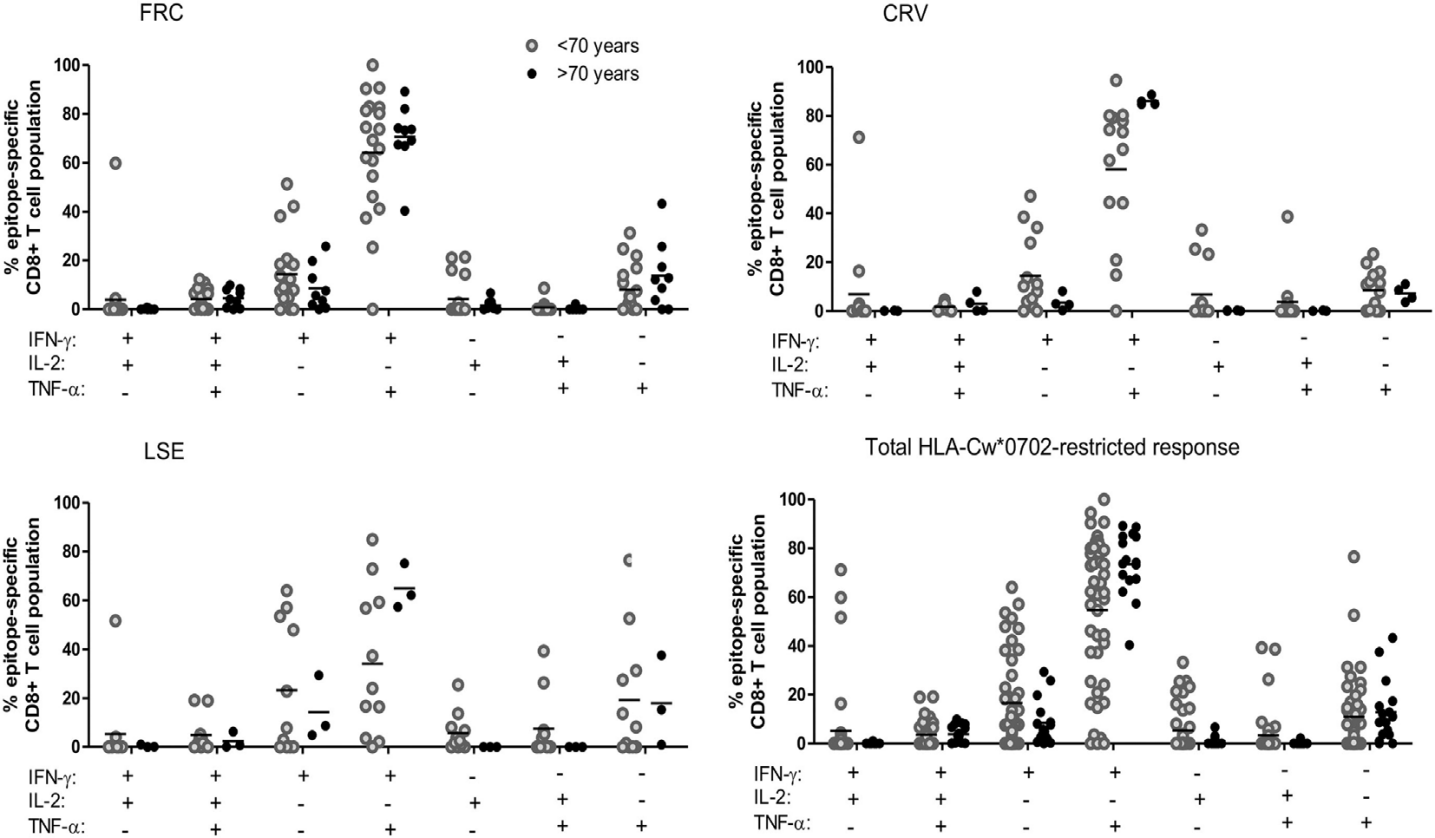
The pattern of co-expression of IFN-γ, TNF-α, and Il-2 cytokines within HLA-Cw*0702-restricted cytomegalovirus-specific CD8^+^ T-cells. Co-expression of the three cytokines was determined within CD8^+^ T-cells following stimulation with peptide. The co-expression of IFN-γ, IL-2, and TNF-α by the total FRC (top left), CRV (top right) and LSE-specific CD8^+^ T-cell populations (bottom left) and total HLA-Cw*0702 CD8^+^ T-cell responses (bottom right) observed within all donors are represented. Donors were assigned into two groups based on age <70 or >70 years. The different combinations of IFN-γ, IL-2, and TNF-α co-expression are indicated on the *x*-axis. Combinations were calculated using the Boolean gating strategy in Kaluza 1.3 software, and percentages were generated using the funky cells software (36). The Boolean gating strategy is provided in Figure S8 in Supplementary Material. Statistical significance was tested by Kruskal–Wallis test with Dunn’s corrections applied in GraphPad Prism 6.

Expression of polyfunctional capacity by CD8^+^ T-cells is associated with enhanced antiviral activity (40, 41). Therefore, we next examined the pattern of cytokine co-expression by HLA-Cw*0702-restricted CD8^+^ T-cells in younger and older donors (gating strategy Figure S8 in Supplementary Material). The majority of T-cells exhibited simultaneous production of both IFN-γ and TNF-α, and this proportion increased from 54.7% in younger donors to 73.5% in older people although this did not reach statistical significance (Figure 4). An important observation was a decrease in T-cells secreting either IFN-γ or TNF-α alone and a notable reduction in the proportion of IL-2 secreting cells (Figure 4). This is likely to reflect the acquisition of an increased polyfunctional effector profile during differentiation and clonal expansion in association with age (42, 43).

**Figure 5:**
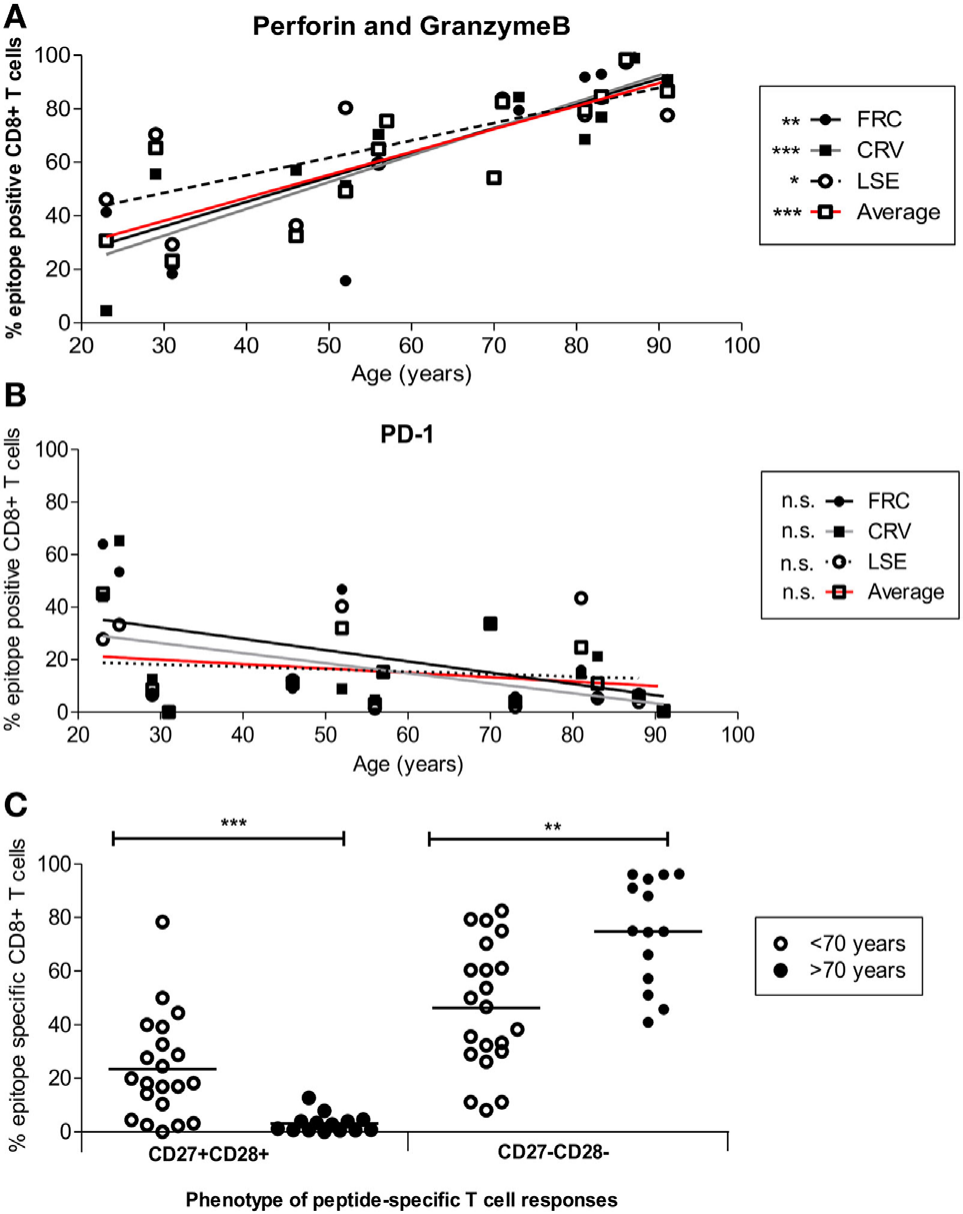
Cytotoxic and differentiation phenotype of HLA-Cw*0702-restricted cytomegalovirus (CMV)-specific CD8^+^ T-cells in relation to age. **(A)** HLA-Cw*0702-restricted CMV-specific CD8^+^ T-cells were identified after peptide stimulation followed by intracellular cytokine staining for TNF-α. Intracellular dual expression of perforin and granzymeB was then determined by flow cytometry. *R* values: FRC = 0.79, CRV = 0.90, LSE = 0.72, and Average = 0.87. **(B)** CMV-specific cells were identified as in panel **(A)** and examined for surface expression of PD-1. *R* values: FRC = -0.47, CRV = −0.43, LSE = −0.2, and Average = −0.25. Values are shown for each individual peptide response (FRC—black circle and black line; CRV—black squares and gray line; LSE—white circles and broken line) and also for the aggregate values of all HLA-Cw*0702-restricted cells (solid red line). Lines represent linear regression, and statistical significance was obtained by Spearman’s rank correlation in GraphPad Prism 6 (*p < 0.05, **p < 0.01, and ***p < 0.001). **(C)** HLA-Cw*0702-restricted CD8^+^ T-cells were identified as in panel **(A)**. The differentiation status of the epitope-specific CD8^+^ T-cell responses was then analyzed using CD27 vs CD28 expression. Statistical significance was determined by Mann–Whitney test in GraphPad Prism 6 (**p < 0.01 and ***p< 0.001).

Cytotoxic capacity was next determined by the intracellular co-expression of perforin and granzymeB (Figure 5A). Dual expression increased from 18.4, 21.2, and 29.3% of FRC-, CRV-, and LSE-specific T-cells respectively in a 31-year-old donor to 99.2, 99, and 97.3% in a donor of 88 years (Figure 5A). To investigate the potential regulation of cytotoxic cells, we also determined the pattern of PD-1 and CD39 expression that have been associated with an exhausted phenotype on some populations of virus-specific CD8^+^ T-cells (44, 45). PD-1 expression was seen to vary markedly between individuals, with a range of 0.44–65.4%, and the number of PD-1^+^ cells decreased during aging (Figure 5B). CD39 expression was low on CMV-specific CD8^+^ T-cells, in line with previous reports, and this was observed even within donors aged up to 91 years. Of note, this was observed despite the fact that CD39 expression was observed on a large proportion of the total CD8^+^ T-cell population (Figure S7 in Supplementary Material).

Loss of CD27 and CD28 is observed on late-differentiated memory cells, and the pattern of CD27 and CD28 co-expression was therefore used to contrast the pattern of differentiation of CD8^+^ T-cells between young and older individuals (46). Interestingly, CD27 and CD28 expression was lost on the majority of HLA-Cw*0702-restricted T-cells within older individuals (ranges 41.0–96.3%) whereas CD27^+^CD28^+^ populations became much less common (Figure 5C).

These data indicate that HLA-Cw*0702*-*restricted CMV-specific CD8^+^ T-cells become more differentiated during aging but remain highly functional and do not develop evidence of cellular “exhaustion.”

### HLA-Cw*0702-Restricted CMV-Specific CD8^+^ T-Cells Exhibit Very High Avidity with Age and Recognize Infected Cells for up to 72 h after Infection

The avidity of CD8^+^ T-cells for their HLA-peptide target can be used as a correlate of antiviral efficacy (47) and is an important determinant of memory inflation within CMV-specific CD8^+^ T-cells (48–50).

**Figure 6:**
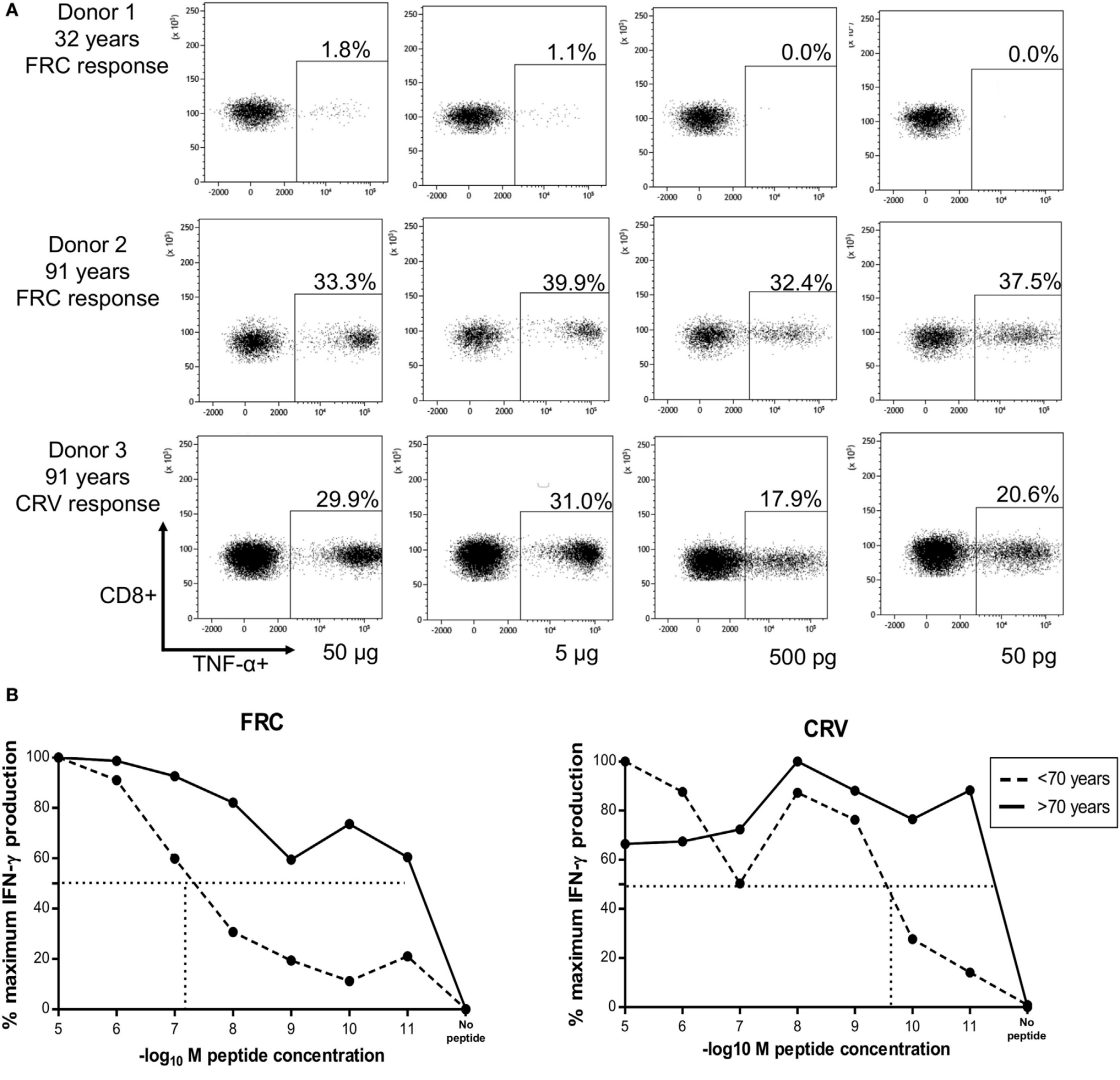

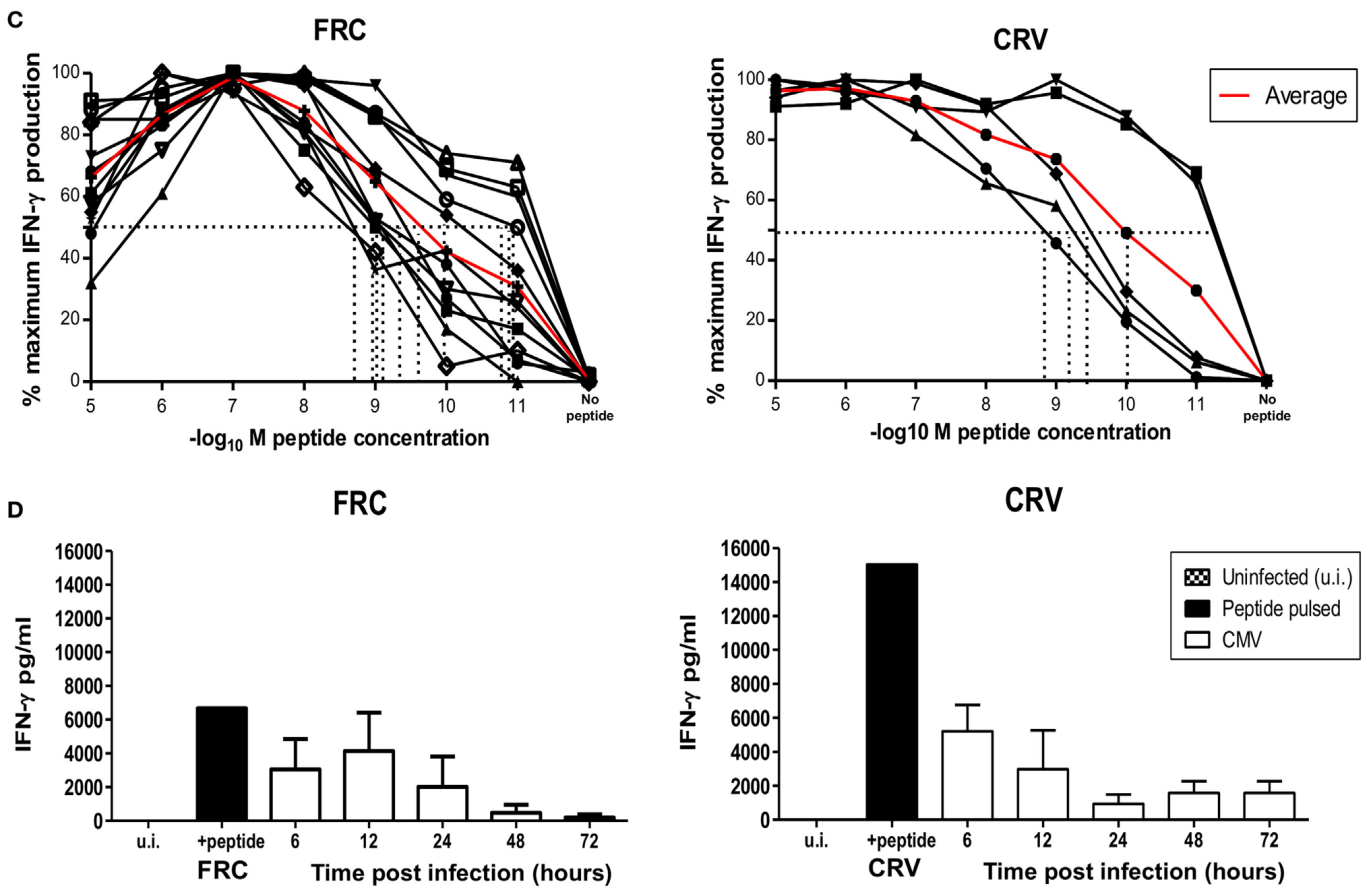
HLA-Cw*0702-restricted CD8^+^ T-cells have high avidity and recognize cells in the immediate-early time point of viral infection. **(A)** TNF-α production by PBMCs in response to HLA-Cw*0702-restricted peptide gradient. 50,000 PBMCs were incubated with peptide concentration of 50 pg (10^−11^) to 50 µg (10^−5^) in the presence of brefeldin A followed by intracellular cytokine staining for TNF-α. The EC_50_ was defined as the peptide concentration that induced the 50% maximal TNF-α production by HLA-Cw*0702-restricted CD8^+^ T-cells as a percentage of the total CD8^+^ T-cell population. Percentages represent the percentage of the total CD8^+^ T-cell pool responding to that concentration of HLA-Cw*0702-restricted peptide. The two highest (50 and 5 µg) and two lowest (500 and 50 pg) peptide concentrations are shown as representative examples. **(B)** Functional avidity of HLA-Cw*0702-restricted cytomegalovirus (CMV)-specific PBMC. 50,000 PBMCs from three young (32, 46, and 56 years) and three elderly donors (83, 91, and 91 years) were incubated with peptide concentrations of 50 pg (10^−11^) to 50 µg (10^−5^), and the EC_50_ was defined as in panel **(A)**. This was determined within GraphPad Prism 6 by applying a sigmoidal dose response curve. Percentages represent the percentage of the total CD8^+^ T-cell pool responding to that concentration of HLA-Cw*0702 peptide. Younger donors (<70 years) are represented by dashed lines, and older donors (>70 years) represented by solid black lines. Lines represent the average percentage of PBMCs responding to that peptide gradient (FRC—left graph and CRV—right graph) within *n* = 3 donors per age group. **(C)** Functional avidity of HLA-Cw*0702-restricted CMV-specific CD8^+^ T-cell clones. CD8^+^ T-cell clones were isolated by limiting dilution analysis from HLA-Cw*0702^+^ CMV-seropositive donors. 1,000 CD8^+^ T-cell clones were cocultured for 16 h with 5 × 10^4^ peptide-pulsed LCL loaded with a peptide concentration gradient of 50 pg (10^−11^) to 50 µg (10^−5^). CD8^+^ T-cell activation was analyzed by IFN-γ ELISA. *n* = 12 clones for FRC and *n* = 5 for CRV. The EC_50_ was determined as in panel **(B)**. The red line represents the average EC_50_ avidity of the CD8^+^ T-cell clones. **(D)** Time course of recognition of HLA-Cw*0702-restricted CMV peptides during viral infection. The presentation of HLA-Cw*0702-restricted peptides during CMV infection was determined by recognition by virus-specific T-cell clones following infection of MRC5 fibroblasts (HLA-A2^+^HLA-Cw*0702^+^) *in vitro*. 10,000 CD8^+^ T-cells were cocultured with the infected fibroblasts, and IFN-γ ELISA was used to measure peptide recognition at the indicated time points. Left graph = FRC-specific T-cell clones and right graph = CRV-specific T-cell clones. *n* = 3–6 per time point and *n* = 3 clones tested per specificity. ui, uninfected cells; peptide, target cells pulsed with cognate peptide. Target cells were infected with *AD169* virus at an MOI of 5. The peptide-pulsed control bar represents the average of all experiment replicates.

We next analyzed the functional avidity of HLA-Cw*0702 CD8^+^ T-cells within donor PBMC through measurement of the peptide concentration that induced 50% maximal cytokine production (EC_50_). PBMC from three young (<70 years) and three elderly (>70 years) donors was incubated with peptide for 16 h followed by detection of intracellular TNF-α (Figure 6A). Peptide-specific CD8^+^ T-cells from elderly donors were found to display higher avidity than those from younger donors (average EC_50_ of younger donors 0.5 µg or 10^−7.1^ log_10_*M* FRC and 0.00125 µg or 10^−9.7^ log_10_*M* CRV, Figure 6B) (Figure 6A).

12 FRC-specific and 5 CRV-specific CD8^+^ T cell clones were also generated, and functional avidity was assessed in these cells. EC_50_ values ranged between 0.5 and 50 pg (10^−9^–10^−11^ log_10_*M*), with an average of 5 pg (10^−10^ log_10_*M*), demonstrating very high clonal avidity for both FRC-specific and CRV-specific clones (Figure 6C).

We also assessed the time course of peptide recognition by HLA-Cw*0702-restricted T-cells through incubation of FRC-specific and CRV-specific T-cell clones with virus-infected target cells. T-cell recognition peaked at 6–12 h after infection and was retained until 72 h (Figure 6D). HLA-Cw*0702-restricted T-cells therefore have the capacity to recognize their cognate IE peptide epitopes very early during viral infection. These findings suggest that the combination of high avidity and extended period of peptide recognition may serve to drive the relative expansion of HLA-Cw*0702-restricted CD8^+^ T-cells during viral reactivation events.

## Discussion

Virus-specific CD8^+^ T-cells restricted by HLA-C have received considerably less investigation than responses restricted by HLA-A or HLA-B. Here we have shown that CD8^+^ T-cells specific for peptides derived from proteins expressed in the IE phase of CMV replication and restricted by HLA-Cw*0702 elicit potent CD8^+^ T-cell responses. Moreover, these accumulate markedly with age such that they represent over half of the CD8^+^ T-cell repertoire in older people. As such, we believe these to be the largest CD8^+^ T-cell responses ever reported in the human immune system.

Cytomegalovirus infection is most commonly acquired during the first year of life, and although infection can also be acquired in adulthood, it is likely that most of the majority of our donors had carried chronic infection for many decades. Importantly, we were able to detect an increase in the magnitude of CD8^+^ T-cell responses within individual donors over time indicating that “memory inflation” was not simply a cohort effect and is, to the best of our knowledge, the first prospective demonstration of memory inflation within humans. A range of studies suggest that memory inflation is driven by an increase in CMV viral load and it was noteworthy that viral copy number increased from 200 to 400 copies/25 ng DNA from CD14^+^ monocytes within a period of 5 years in our oldest donor aged 91 years. This coincided with an increase in the FRC-specific CD8^+^ T-cell pool from 2.7 to 37%, indicating that CD8^+^ T-cell memory inflation was proportionally much greater than the increase in viremia. As such, the quantitative relationship between peripheral viral load and CMV-specific CD8^+^ T-cell response is unclear, and one factor may relate to the relative presentation of viral antigen within secondary lymphoid tissue.

The magnitude of the HLA-Cw*0702-restricted CMV-specific immune response is further support for the idea that virus-specific cells explain the association between persistent CMV infection and an increase in the size of the total memory T-cell pool (51) and inversion in the CD4:CD8 ratio (52). However, the magnitude of memory inflation varied within individual donors in line with previous studies. The determinants of memory inflation are not yet clearly established but could include the magnitude of the initial viral inoculum (53) and the frequency and magnitude of episodes of subclinical viral reactivation. Indeed, subclinical episodes of viral reactivation can be suppressed by T-cells before infectious virions are produced (54, 55) and abortive viral reactivation with expression of IE genes in the absence of early or late genes is observed in murine CMV infection (56). It is therefore significant that the three peptides that drive HLA-Cw*0702-restricted memory inflation are all derived from proteins synthesized in the IE phase of viral replication (57). In this regard, it is noteworthy that we observed only modest non-inflationary CD8^+^ T-cell responses to the HLA-Cw*0702-restricted UL33-derived SYR peptide that is generated in the late phase of replication. Several studies have now identified that the expression kinetics of CMV peptides determines their immunodominance and capacity to drive CD8^+^ T-cell inflation (57–60). Indeed, HSV-1 epitopes induce inflationary CD8^+^ T-cell populations when expressed under the influence of CMV promoters with IE kinetics whereas those linked to non-IE promoters do not (57).

HLA-C alleles are relatively protected from HLA downregulation following viral infection (20, 31, 32), and this is likely to be a factor driving the memory inflation observed in our work (31, 61). Proteomic analysis of CMV-infected fibroblasts revealed downregulation of HLA-A and HLA-B proteins by 24 h while HLA-C expression started to decline only at 72 h (32). An important observation is that it was the HLA-Cw*0702 allele that was specifically associated with substantial memory inflation. The HLA-Cw*0702 protein is relatively resistant to downregulation by US2-11 proteins due to polymorphism within the α3 domain and C terminus (30). Interestingly, expression of HLA-Cw*0702 is also relatively protected following adenovirus infection where it plays an important role in engaging the inhibitory protein KIR2DL3 on NK cells (62). As such, this allele may play a particularly critical role in suppression of NK recognition during CMV infection and could permit the generation of substantial cytotoxic HLA-Cw*0702-restricted T-cell responses. However, we cannot speculate that relative resistance of HLA-Cw*0702 to US2-11 proteins is the sole factor and other HLA-C alleles may also exhibit similar properties. We also investigated if the expression of HLA-C increases with age and did observe a non-significant trend in the intensity of DT9-staining on monocytes in older people (data not shown). As such, this may be an additional factor that drives HLA-C-restricted CD8^+^ memory inflation although the full biological significance is currently unclear.

A further noteworthy characteristic of HLA-Cw*0702-restricted T-cells is their very high avidity for cognate peptide-HLA, and this was seen to increase substantially between younger and older donors. Functional avidity is critical in determining clonal dominance of peptide responses during chronic infection (49, 63), and the lower surface expression of HLA-C expression compared with HLA-A or HLA-B may act to select for T-cells with high-affinity TCR. Furthermore, immunodominance of CD8^+^ T-cells is determined by competition for cognate peptide on target cells (49, 63), and expanded HLA-Cw*0702-restricted responses might therefore be expected to limit the expansion of T-cells restricted through HLA-A or HLA-B alleles. We did indeed find that HLA-Cw*0702-restricted responses were larger than the combined response to a range of immunodominant peptides presented through HLA-A and HLA-B alleles (Figure S6 in Supplementary Material). Interestingly, expanded populations of low-avidity HLA-A-restricted CMV-specific CD8^+^ T-cells accumulate in many older individuals (64) and may perhaps indicate a relative shift in the importance of HLA-A/B and HLA-C-restricted T-cells in the control of CMV reactivation across the human life course. An extension of this argument might be that persistent viral infections have served to act as an important selective pressure for the evolution of HLA-C to maintain lifelong viral control.

Frequent stimulation of viral-specific CD8^+^ T-cells by chronic exposure to antigen can lead to cellular dysfunction and “exhaustion” (39). Despite this, CMV-specific CD8^+^ T-cells generally retain functional activity within older donors (41, 65), and this pattern was observed for HLA-Cw*0702-restricted responses which exhibited a potent Th1 cytokine and cytotoxic phenotype. Indeed, these phenotypic features increased further during aging with no increase in PD-1 (66) or CD39 expression (45).

Adoptive therapy with T-cells specific for peptides restricted by HLA-A or HLA-B alleles is effective in control of viremia in immune suppressed individuals, and the unique properties of HLA-Cw*0702-restricted CD8^+^ T-cells suggest that they could also have potential for immunotherapy (3, 67–69). The HLA-Cw*0702 allele has a population prevalence of 15–20% in different ethnic groups, and IE-specific T-cells are effective in controlling CMV reactivation events in both humans and mouse models (70, 71). Furthermore, CMV reactivation following hemopoietic stem cell transplantation leads to upregulation of HLA-C expression on many different cell types and the potential utility of HLA-C-restricted T-cells for cellular immunotherapy therefore deserves further investigation (72).

Given the unprecedented magnitude of HLA-Cw*0702-restricted CD8^+^ T-cells in older individuals, it is also important to consider if this may lead to any detrimental effects such as immune senescence (73), vascular disorders (74), and reduced survival (52, 75–77). As such, although expansion of HLA-C-restricted CD8^+^ T-cells appears effective in controlling clinical CMV reactivation in older people, it is also possible that they may also play a detrimental role in some settings.

In conclusion, our work demonstrates that CMV-specific CD8^+^ T-cells that are restricted by HLA-Cw*0702 expand enormously during healthy aging and come to dominate the memory CD8^+^ T-cell pool in older people. These findings contribute to growing appreciation of the importance of HLA-C in the control of chronic viral infections and indicate that further attention should be given to the physiological and therapeutic role of HLA-C-restricted CD8^+^ T-cells.

## Ethics Statement

The recruitment of healthy participants was approved by the Solihull ethics committee, study reference; 14/WM/1254. All participants were adults aged over 18 years, and written and informed consent was taken before participation in accordance with the Declaration of Helsinki. This donor cohort included samples from healthy laboratory personnel, healthy donors from the NHS Blood Transfusion Service and healthy older adults recruited from the “Birmingham 1000 Elders cohort.”

## Author Contributions

LH contributed to the design, acquisition, and interpretation of manuscript data. In addition, LH contributed to manuscript preparation, manuscript proofing, and submission of the final version. AP contributed to the design, acquisition, and interpretation of data. JZ contributed to the manuscript preparation and manuscript proofing of the final version. HP contributed to the acquisition of manuscript data. SR contributed to the acquisition of manuscript data by identifying the novel CMV peptides. PM contributed to the manuscript preparation, manuscript editing, and manuscript proofing of the final version.

## Conflict of Interest Statement

The authors declare that the research was conducted in the absence of any commercial or financial relationships that could be construed as a potential conflict of interest.
