# Supplementary material for: Cytomegalovirus-Specific T Cells Restricted by HLA-Cw*0702 Increase Markedly with Age and Dominate the CD8+ T-Cell Repertoire in Older People
Source: Front Immunol. 2017 Dec 11;8:1776. doi: 10.3389/fimmu.2017.01776 (PMC5732243; doi:10.3389/fimmu.2017.01776)
Supplement: Supplementary file 1 [file Data_Sheet_1.docx]

Supplementary Material

Cytomegalovirus-specific T cells restricted by HLA-Cw*0702 increase markedly with age and dominate the CD8+ repertoire in older people

Louise Hosie^1*^, Annette Pachnio^1^, Jianmin Zuo^1^, Hayden Pearce^1^, Stanley Riddell^2^ and Paul Moss^1^

*** Correspondence:** Corresponding Author: [l.c.hosie@bham.ac.uk](mailto:l.c.hosie@bham.ac.uk)

**
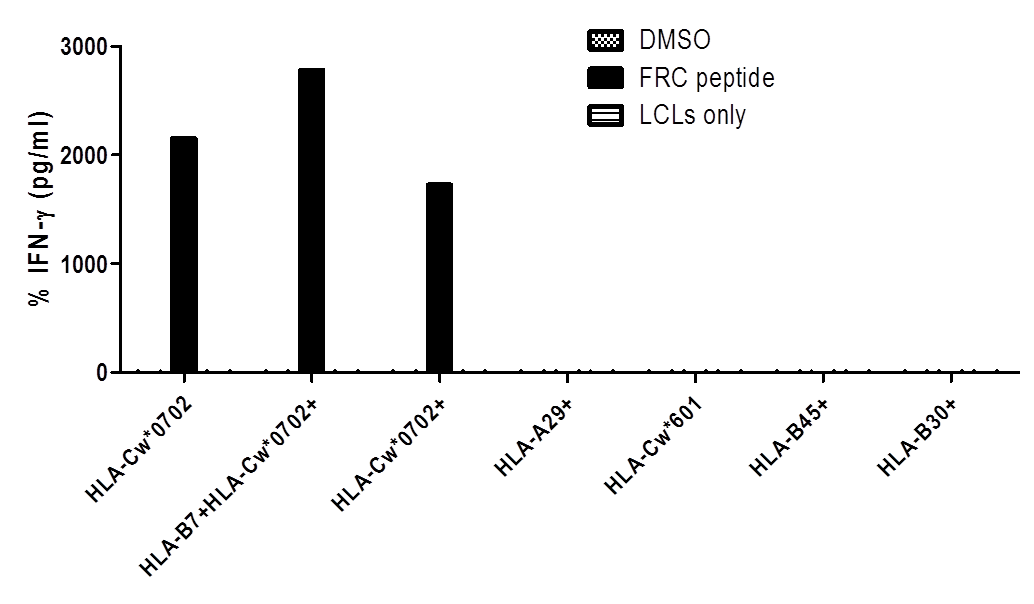
**

**Supplementary Figure 1 Determining the HLA-Cw*0702-restriction of a FRC-specific CD8+T-cell clone.**

An FRC-specific clone was obtained from a seropositive donor of 49 years with the HLA type - *HLA-A29^+^/B7^+^/B30^+^/B45^+^/Cw601^+^/Cw0702^+^*. To determine the HLA-restriction of the CD8+T-cell clone and subsequently the peptide, the clone was co-cultured O/N with 2 x 10^4^ FRC peptide loaded LCL lines that were positive for one of the HLA alleles within the HLA type of the donor that the clone was isolated from. The exclusion to this was the dual *HLA-B7^+^/Cw*0702^+^* LCL line to discriminate between the two alleles as they are commonly inherited together by linkage disequilibrium. The positive recognition of the FRC: HLA complexes was determined by IFN-γ ELISA following the O/N co-culture.

**
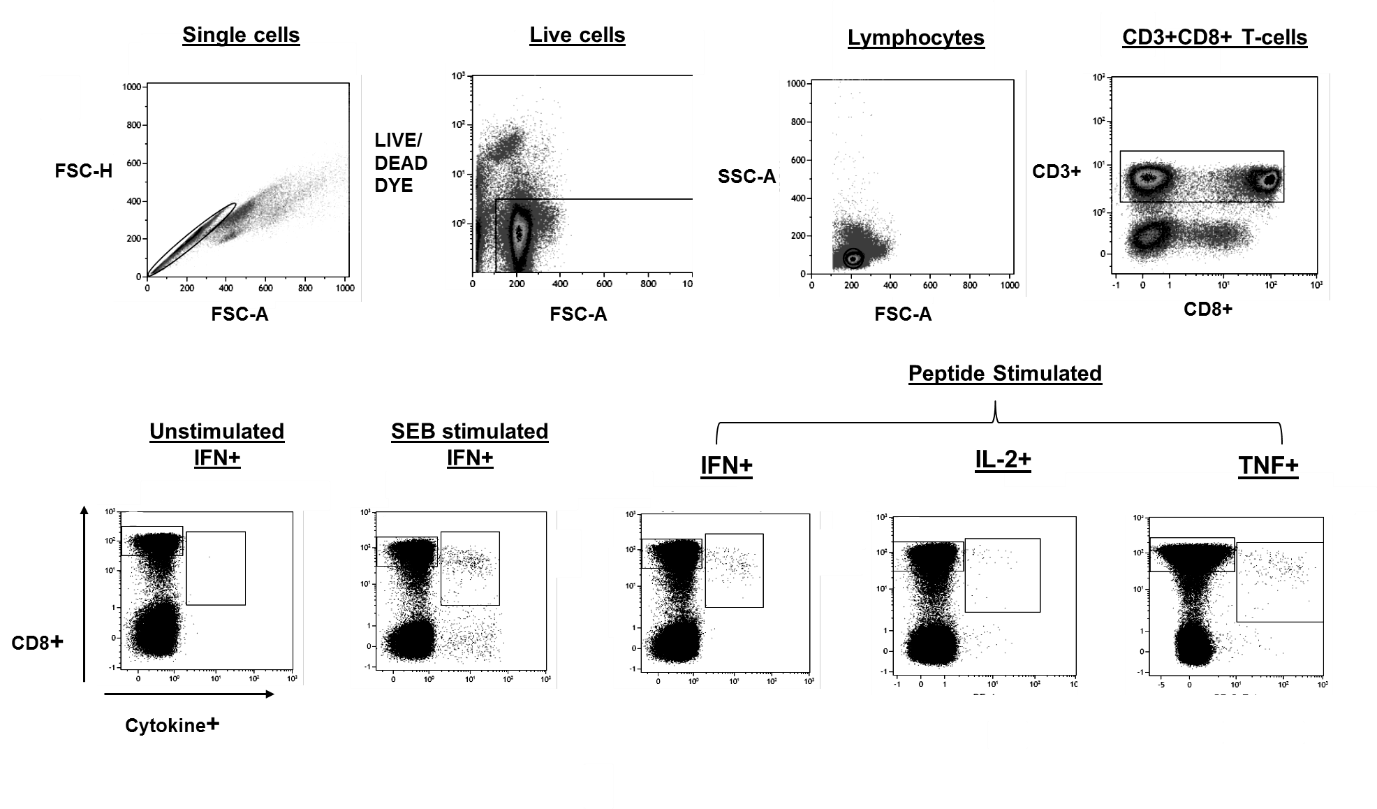
**

**Supplementary Figure 2 Gating strategy for identifying activated CD8+T-cells in response to HCMV peptide incubation**

**(A) Identifying activated CD8+T-cells in response to peptide stimulation by intracellular cytokine staining.** Cells were gated sequentially within Kaluza 1.3 software in the following hierarchal manner; single cells, live cells (LIVE/DEAD Fixable Dead Cell Stain, Invitrogen), lymphocytes, CD3+CD8+ lymphocytes and finally CD8+ lymphocytes vs the cytokine in question to identify those producing the cytokine. Example dot plots include (left to right) unstimulated negative control, SEB stimulated positive control and IFN-γ/IL-2/TNF-α production after HCMV peptide stimulation. The peptide stimulated tubes provided in this example are stimulated with the FRC peptide.

**
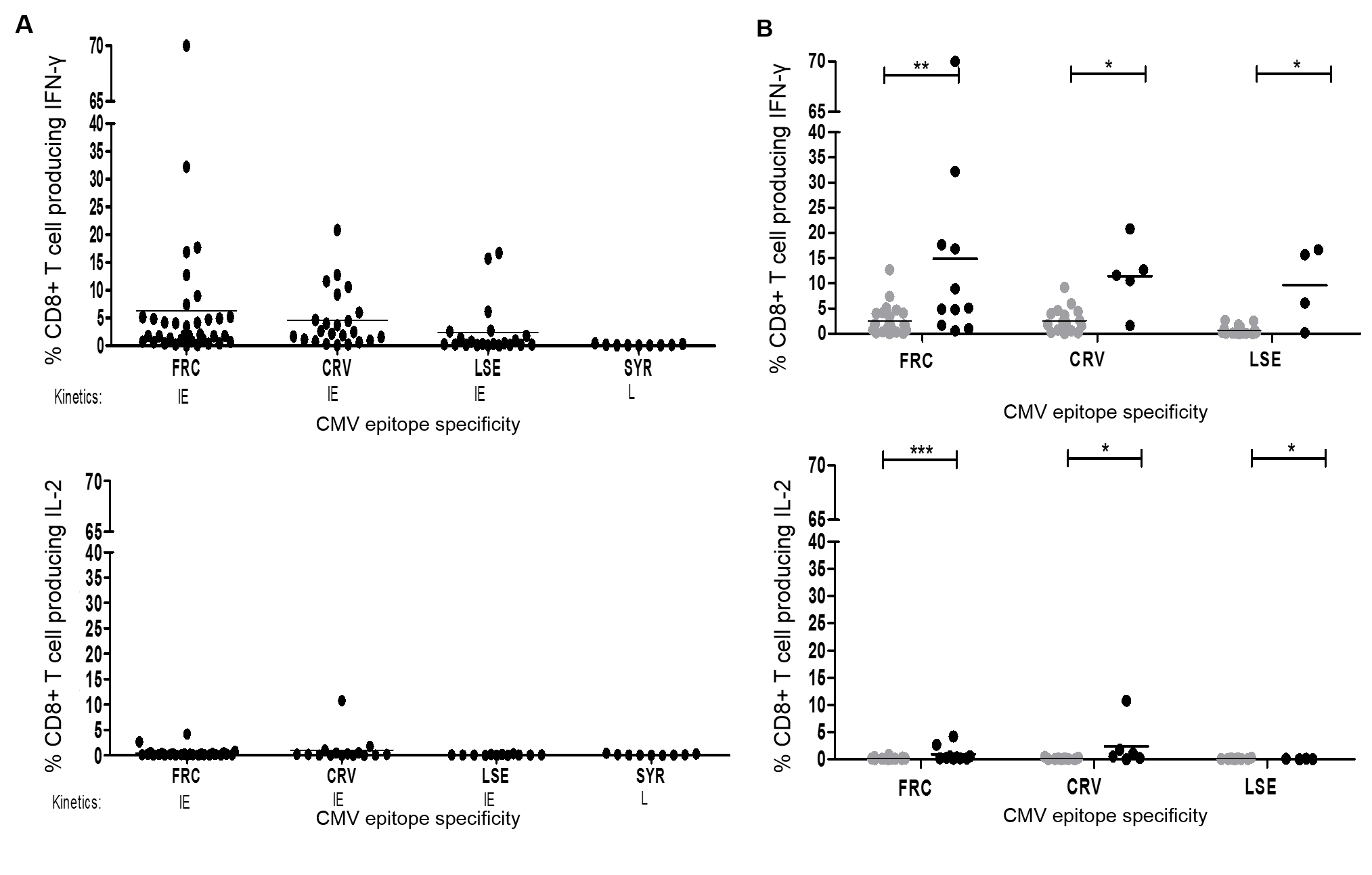
**

**Supplementary Figure 3 IFN-γ and IL-2 production by CD8+T cells in HCMV seropositive donors after HLA-Cw*0702-restricted peptide epitope stimulation**

**(A) The percentage of CD8+ T-cells producing IFN-γ and IL-2 in response to HLA–Cw*0702 restricted HCMV peptide epitopes.** Specific CD8+T-cells were identified after 6 hours peptide stimulation (1µg/ml final concentration) and intracellular cytokine staining to identify IFN-γ (top) and IL-2 (bottom) production. Each symbol represents the percentage of an individual donor’s total CD8+T-cell pool producing that cytokine in response to the peptide in question (Table 1). Lines represent mean. n = 8-32 positive responses detected per peptide epitope tested. **(B) The percentage of CD8+ T-cells producing IFN-γ and IL-2 in response to *HLA–Cw*0702*-restricted HCMV peptide epitopes between donors <70 years of age vs donors >70 years of age** Specific CD8+ T-cells were identified after 6 hours peptide stimulation (1µg/ml final concentration) and intracellular cytokine staining to identify IFN-γ (top) and IL-2 (bottom) production. Lines represent mean. n = 8-32 positive responses detected per peptide epitope tested. Younger donors < 70 years of age are represented by grey circles and older donors >70 years of age are represented by black circles.


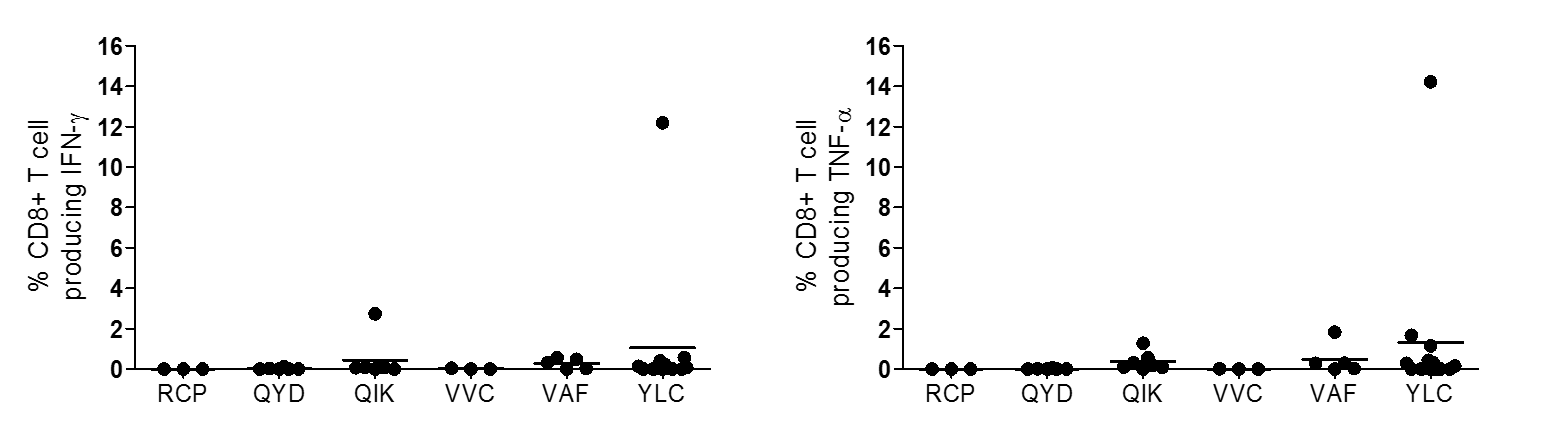


**Supplementary Figure 4 IFN-γ and TNF-α production by CD8+ T-cells in HCMV seropositive donors after stimulation with HLA-C peptide epitopes not restricted by HLA-Cw*0702**

Specific CD8+ T-cells were identified after 6 hours peptide stimulation (1µg/ml final concentration) and intracellular cytokine staining to identify IFN-γ (left) and TNF-α (right) production. Each symbol represents the percentage of an individual donor’s total CD8+ T-cell pool producing that cytokine in response to the peptide in question (Table 1). Lines represent mean. n = 3-13 donors tested per peptide epitope tested.

***
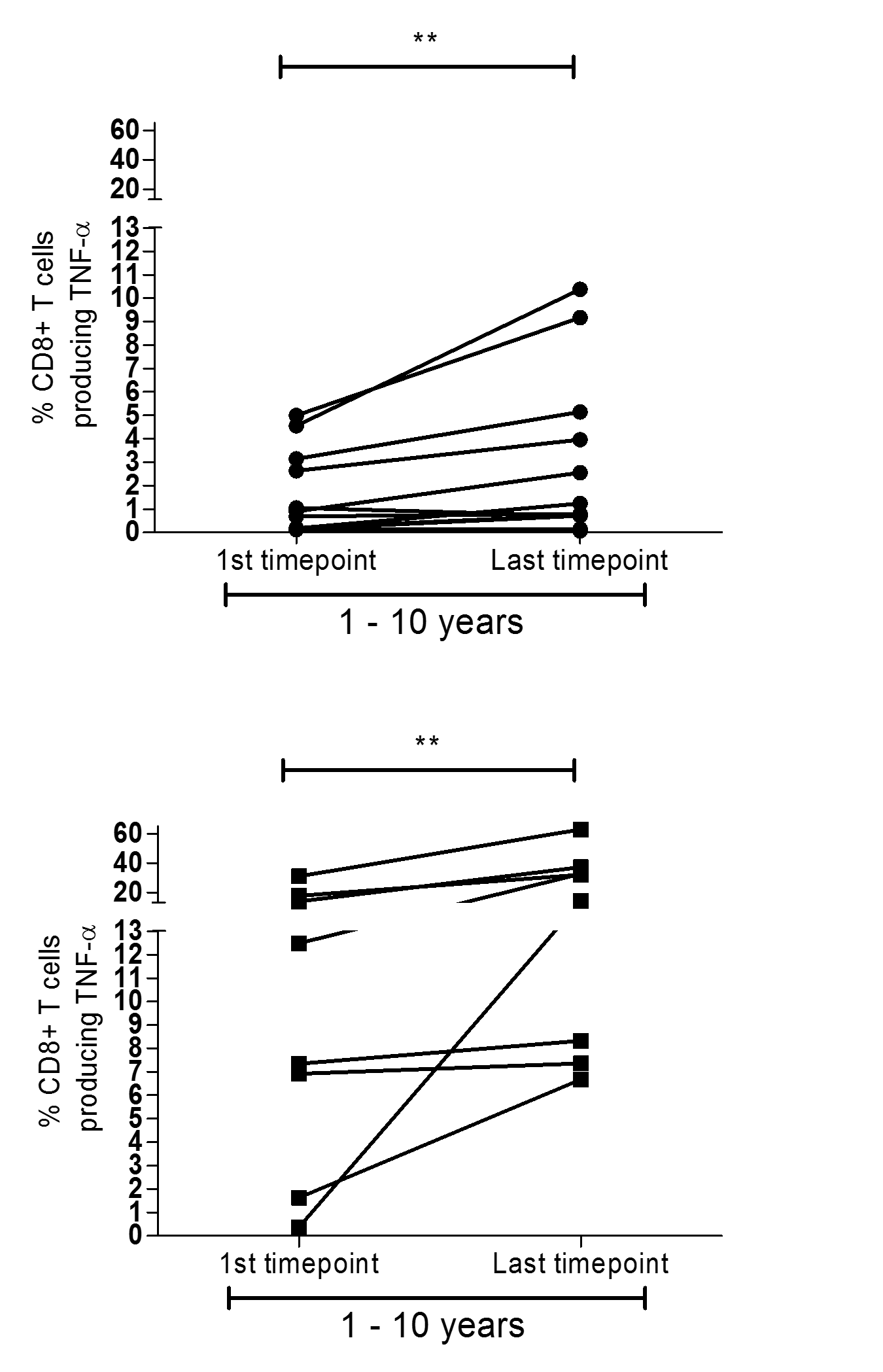
***

**Young donors (<70 years)**

**Older donors (>70 years)**

**Supplementary Figure 5 - Memory inflation of HLA-Cw*0702-restricted T cell responses is seen within individual young vs elderly donors.**

HLA-Cw*0702-specific CD8+ T-cell responses were followed within young (<70 years, top graph) and elderly donors (>70 years, bottom graph) using blood samples taken between 1 and 10 years apart. Figure shows the TNF-α response at the initial and end time points**.** Percentages represent the TNF-α response to HLA-Cw*0702-restricted peptide as proportion of the total CD8+ T-cell repertoire. Statistical significance was determined by a paired Wilcoxon T test.

***
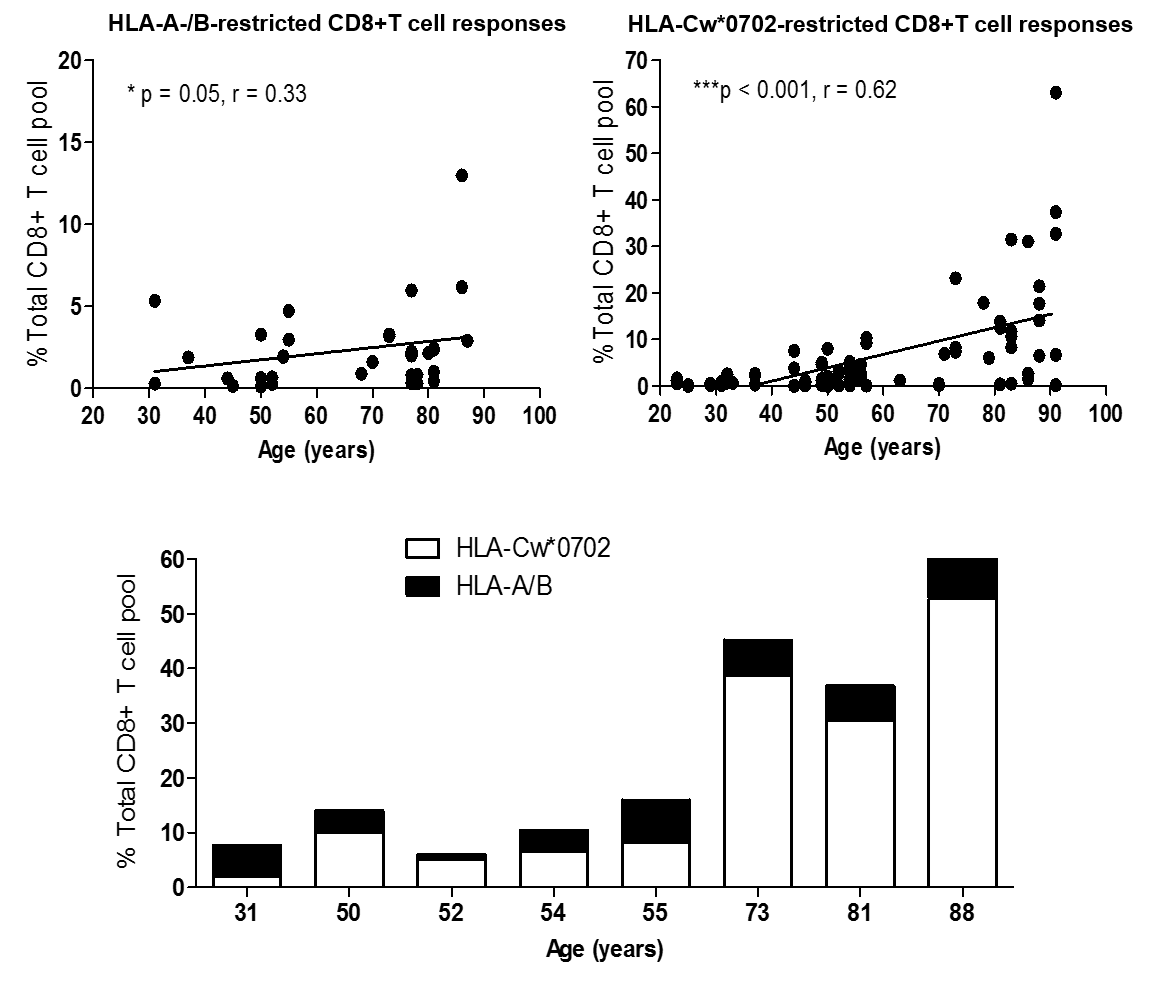
***

**A.**

**B.**

**C.**

**Supplementary Figure 6 HLA-Cw*0702-restricted CD8+ T-cell populations are immunodominant over HLA-A/-B restricted CD8+ T-cell populations within the same seropositive individuals**

Donors that were screened for responses to HLA-Cw*0702-restricted peptides were subsequently screened for CD8+T-cell responses to HLA-A/-B restricted peptides derived from the pp65, pp50 and IE-1 proteins (HLA-A1- VTE , HLA-A2- NLV and VLE and HLA-B7-restricted RPH and TPR ). Specific CD8+ T-cells were identified after 6 hours peptide stimulation (1µg/ml final concentration) and intracellular cytokine staining to identify TNF-α production. **(B) CD8+ T-cell responses towards, HLA-A, -B and –Cw*0702-restricted CMV CD8+ T-cell peptides correlated with age.** The CD8+T-cell responses towards the HLA-A-/-B-restricted peptides (Left graph) vs HLA-Cw*0702-restricted FRC, CRV and LSE peptides (Table 1)(Right graph) were identified and correlated with the age of the donor cohort. **(C) Total magnitude of HLA-A/B restricted CMV CD8+ T-cell responses vs ‘inflationary’ HLA-Cw*0702-restricted CD8+ T-cell responses in donors that exhibit individual CD8+ T-cell responses to each of the FRC, CRV and LSE peptides.** The black bars represent the aggregated sum of the TNF-α production by HLA-A-/-B-restricted CD8+ T-cell responses towards the HLA-A1- VTE, HLA-A- NLV and VLE and HLA-B7-restricted RPH and TPR peptides, identified as in (A). The white bars represent the aggregated sum of the TNF-α production by HLA-Cw*0702-restricted FRC-, CRV- and LSE-specific CD8+ T-cell responses within the same individual donors.

**
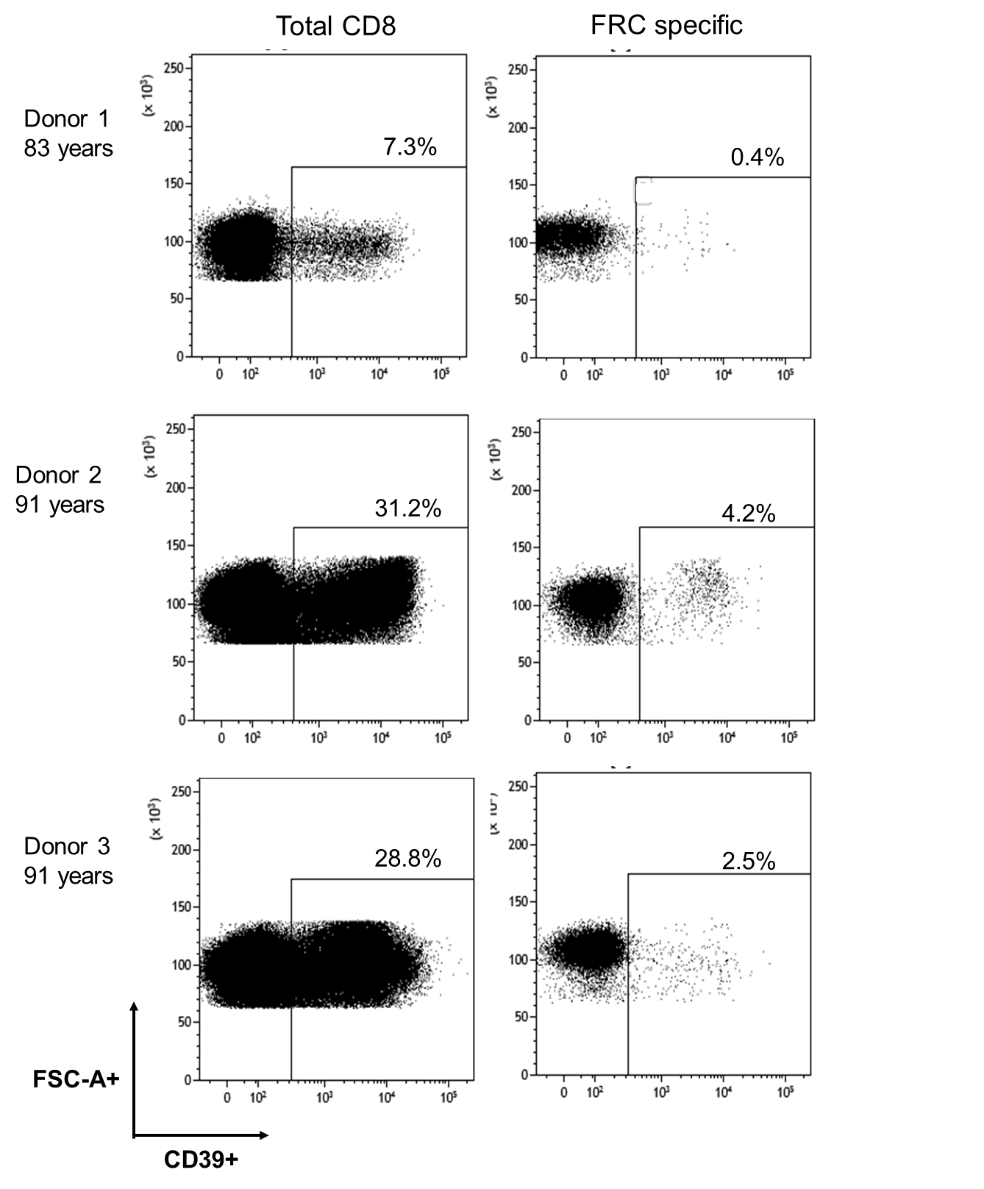
**

**Supplementary Figure 7 CD39 expression on HLA-Cw*0702 FRC-specific CD8+ T-cell responses within three older donors aged >70 years old**

The largest FRC-specific HLA-Cw*0702-restricted CD8+ T-cell responses detected within the oldest donors were phenotyped for CD39 expression (Biolegend, α-PE) after 6 hours peptide stimulation (1µg/ml final concentration). CD39 expression on total CD8+ T-cells is provided in the left hand column and expression on FRC-specific CD8+ T-cells on the right hand column. The age of the donor is provided on the left hand side of the plots.

**
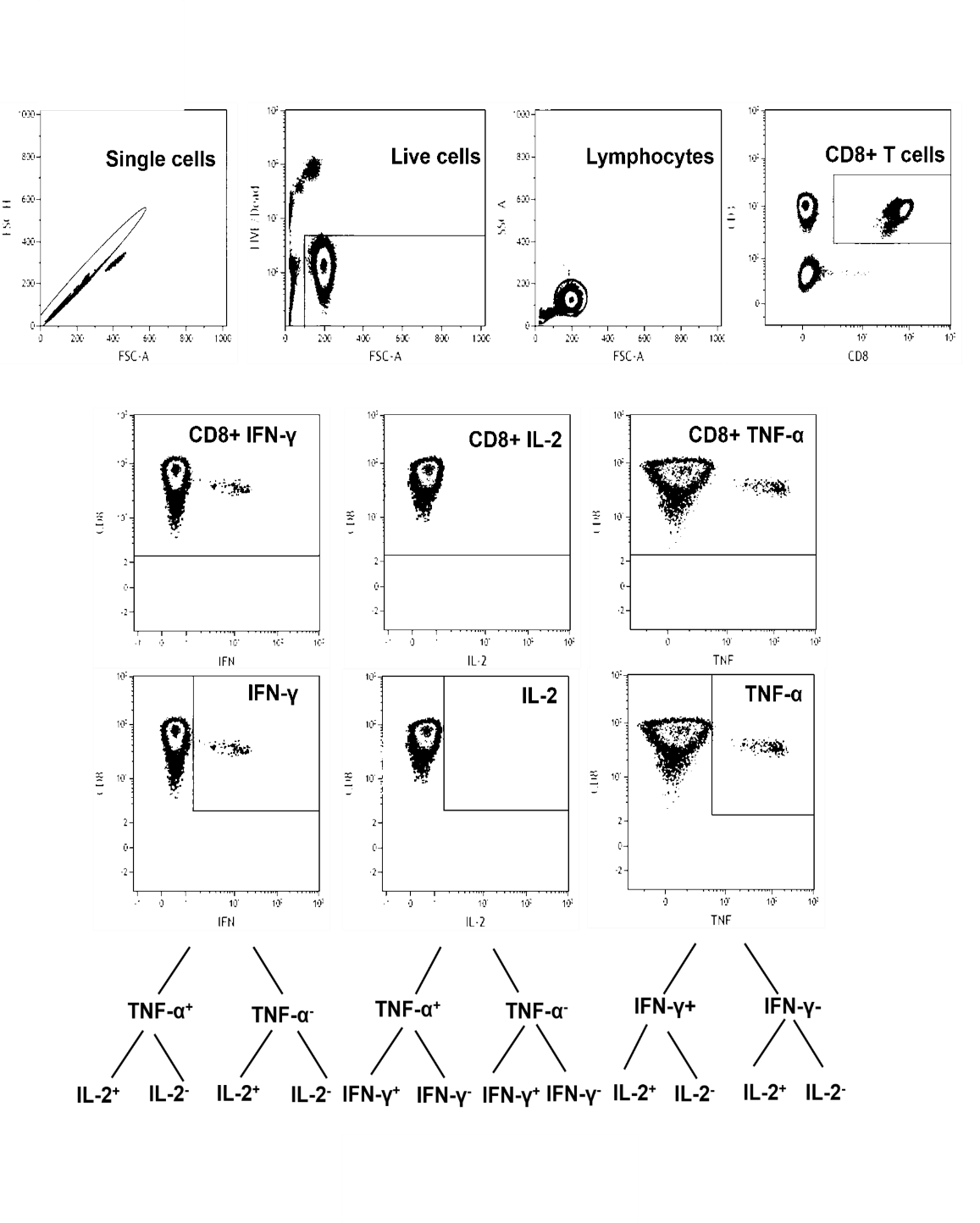
**

**Supplementary Figure 8 – Gating strategy for identifying cytokine polyfunctionality using Boolean gating within Kaluza 1.3 software and Funky cells software.**

Single, live CD8+ lymphocytes were hierarchically gated upon. Individual gates for CD8+T-cells including the three separate cytokine functions were then placed on the CD8+T-cells. A positive gate for each three cytokine function was then also placed. Within Kaluza 1.3 software, Boolean gates for each combination of cytokine production were manually generated and the numbers of cells within those gates generated by the Kaluza software. Within the data, the IFN-γ^-^IL-2^-^TNF-α^-^ combination was excluded as the combination is negative in all cases due to the epitope-specific cells being identified via cytokine production. The numbers of cells within each combination were then inputted into ‘Funkycell’ software. The resulting polyfunctional output including percentage of cells within each combination were generated into graphical format within Graphpad Prism 6.
